# Hysteresis in Audiovisual Synchrony Perception

**DOI:** 10.1371/journal.pone.0119365

**Published:** 2015-03-16

**Authors:** Jean-Rémy Martin, Anne Kösem, Virginie van Wassenhove

**Affiliations:** 1 Université Paris VI (UPMC), Institut d’Étude de la Cognition (IEC) & Institut Jean-Nicod (IJN, ENS-EHESS-CNRS), Paris, France; 2 CEA, DSV/I2BM, NeuroSpin, INSERM, U992, Cognitive Neuroimaging Unit, Univ Paris-Sud, F-Gif/Yvette, France; Monash University, AUSTRALIA

## Abstract

The effect of stimulation history on the perception of a current event can yield two opposite effects, namely: adaptation or hysteresis. The perception of the current event thus goes in the opposite or in the same direction as prior stimulation, respectively. In audiovisual (AV) synchrony perception, adaptation effects have primarily been reported. Here, we tested if perceptual hysteresis could also be observed over adaptation in AV timing perception by varying different experimental conditions. Participants were asked to judge the synchrony of the last (test) stimulus of an AV sequence with either constant or gradually changing AV intervals (constant and dynamic condition, respectively). The onset timing of the test stimulus could be cued or not (prospective *vs*. retrospective condition, respectively). We observed hysteretic effects for AV synchrony judgments in the retrospective condition that were independent of the constant or dynamic nature of the adapted stimuli; these effects disappeared in the prospective condition. The present findings suggest that knowing *when* to estimate a stimulus property has a crucial impact on perceptual simultaneity judgments. Our results extend beyond AV timing perception, and have strong implications regarding the comparative study of hysteresis and adaptation phenomena.

## Introduction

Past experience is known to influence perceptual decisions in two distinct and opposite ways. On the one hand, repeated exposure to the same sensory inputs can lead to adaptation and thus, after-effects. For instance in the classic motion after-affect, after adaptation to a stimulus grating moving in a specific direction, an immobile grating will appear to move in the opposite direction [1]. The perception of an incoming stimulus is thus affected in the direction opposite to the preceding stimulation [1–6]. On the other hand, hysteresis yields persistence effects, namely: repeated exposure to similar sensory inputs allows for the maintenance of a constant percept over time hence in the same direction as the adaptor [7–12]. Both persistence effects and after-effects can simultaneously influence perceptual decisions [13,14] yet numerous reports highlight the dominance of one effect over the other depending on the quality of the stimulus and the task at hand [3,8,9,12,15].

In audiovisual (AV) perception, adaptation has been reported to support most contextual effects, using either Temporal Order Judgment (TOJ) [4,16–18] or Simultaneity Judgment (SJ) [3,19] tasks. In these experiments called “lag adaptation” [18], when participants are adapted to asynchronous AV stimuli with a particular time lag (with either the sound leading the visual stimulus or the visual stimulus leading the sound) time judgments that follow are biased in the direction of the AV lag adaptation. In other words, and consistent with an after-effect, participants have a tendency to perceive AV stimuli as being synchronous after adaptation to asynchronous AV stimuli. This perceptual phenomenon has been interpreted as the ability for the brain to compensate for external and internal transmission delays—i.e. difference in the speeds of sound and light, differences of transduction times and inherent neural conduction delays [20–22]. Interestingly, persistence effects have been reported in unisensory timing perception (e.g. with tactile stimuli [18]) and recently in AV timing, but only when lag adaptation mechanisms were cancelled out by specific experimental manipulations [15]. In other words, when lag adaptation mechanisms were fully operational, they seemed to dominate hysteresis mechanisms in AV temporal perception.

In all AV lag adaptation studies, the adaptation periods consisted in the repetition of AV stimuli with constant or normally distributed AV temporal intervals. Additionally, participants were aware of the arrival of the relevant test stimulus: participants were either cued prior to the presentation of the test stimuli [3,4,16,17,19] or performed synchrony judgments on a trial-by-trial basis, meaning that each ongoing stimulus was a test stimulus [15,18]. However, in hysteresis paradigms stimulation is often dynamically and progressively changed from one state to another, and the timing of the critical change in stimulation is generally kept uncertain. It is either the task of the participant to report the critical change over time (e.g. “Report when the stimulus changes from percept A to B”) [9–12] or judgments are measured *a posteriori* (or retrospectively) (e.g. “Have you perceived A anytime during the sequence?”) [7,8]. Therefore, we conjectured that both the dynamic (progressively decreasing or increasing AV lags) *vs*. constant (fixed AV lags) nature of the adaptation period as well as the prospective *vs*. retrospective nature of the task would be decisive factors in engaging adaptation *vs*. hysteretic mechanisms.

To test this hypothesis, we designed four psychophysics experiments. In each experiment, trials consisted in sequences of AV stimuli and participants had to perform a SJ [23–26] on the last stimulus (test stimulus) of each sequence. In two *Retrospective Tasks* (Experiments 1 and 2), participants did not know when in the sequence the test stimulus would occur. In two *Prospective Tasks* (Experiments 3 and 4), participants were cued when the AV test stimulus would appear. In Experiments 1 and 3, the temporal interval of AV stimuli was progressively decreasing or increasing as the sequence unfolded (Dynamic condition). In Experiments 2 and 4, the temporal interval of AV stimuli was kept constant throughout the adaptation period (Constant condition).

We found that hysteresis could account for the perception of AV simultaneity only in *Retrospective Tasks* (Experiments 1 and 2) independently of the dynamic or the constant nature of the adaptation sequence. In contrast, hysteretic effects vanished in *Prospective Tasks* (Experiments 3 and 4). Our results suggest that hysteresis may not depend on the dynamic vs. constant nature of the AV temporal intervals used during adaptation but may rather implicate participants’ ability to predict when to estimate the synchrony of an AV event. This strongly suggests that individuals engage different perceptual decision strategies as a function of the temporal predictability of the AV test stimulus.

## Materials and Methods

### 1. Participants

In total, 48 participants took part in the study. All participants had normal or corrected-to-normal vision, no known history of hearing problems, and all were naïve as to the purpose of the study. Participants were recruited from the database of the ‘Relais d’information sur les sciences de la cognition’ (RISC). Written informed consents were obtained from each participant and the experiment was conducted in a properly ethical manner in agreement with the Declaration of Helsinki (2008). The present study was specifically approved by the Ethics Committee of the Université Paris Descartes (Paris 5)/Ecole Normale Supérieure (Paris, France). All participants were compensated 10 euros for their participation in the study.

Participants were randomly divided into four groups. Each group performed one of the four experiments. Fourteen participants (mean age = 23.7, range = 21–29, 10 females, 1 left-handed) were recruited to participate in the Experiment 1; 8 participants took part in Experiment 2 (mean age = 27.7, range = 25–30; four females); 13 participants took part in Experiment 3 (mean age = 25; range = 21–30; 7 females); 13 participants took part in Experiment 4 (mean age = 24.5, range = 20–30; 10 females). Two participants in Experiment 3 and one participant in experiment 4 did not finish the experiment and were thus excluded from data analysis.

### 2. Stimuli and experimental design

All participants performed the task while seated in a quiet room approximately 70 cm from the screen (60 Hz refreshing rate). Auditory stimuli were presented via headphones (HD 250 linear II). The auditory stimulus consisted of a 1500 Hz tone pip with a duration of 15 ms and a linear rise and fall time of 5 ms. The visual stimulus was presented on a black background and consisted of a white ring (outer diameter: 3°; inner diameter: 1.7°), which was flashed for the duration of 1 frame (16.7 ms) at the centre of the screen. A white fixation cross was displayed during the whole trial at the centre of the ring.

Each trial consisted of a sequence of 12 successive AV stimuli (the combination of a sound and a flash). The Inter-Stimulus Interval (ISI) between these AV stimuli was of 867 _±_ 133 ms. In all experiments, participants were asked to estimate the simultaneity of the last AV stimulus (test stimulus) of the sequence. There were four possible AV intervals (time lags) between the sound and the flash of the test stimulus (0, 50, 100 and 150 ms). The choice for these test stimulus AV intervals was motivated by unpublished pilot data and by the typical values found in the literature (e.g., [4]). In half of the trials the sound was preceding the flash (sound-leads trials), in the other half it was the flash that preceded the sound (flash-leads trials).

In all four experiments, each test stimulus AV interval (0, 50, 100 and 150 ms) was presented 12 times for each lag-direction condition (sound-leads trials/flash-leads trials) and each sequence condition (Synchronizing/ Desynchronizing in experiments 1 and 3; Asynchronous/ Synchronous in experiments 2 and 4) for a total of 12*4*2*2 or 192 trials per experiment. The total duration of each experiment (divided in 4 blocks of 15 min) was of about one hour.

#### 2.1. Retrospective (Experiment 1 & 2) vs. prospective (Experiment 3 & 4) judgments

In all experiments, participants were instructed to pay attention to the entire sequence of AV stimuli. Participants were asked to judge the timing of the last AV (test) stimulus in the sequence, and that the timing of the test stimulus could differ from its preceding stimuli.

In Experiment 1 and Experiment 2, participants had to judge *retrospectively* the synchrony of the test stimulus: participants were not told the number of AV stimuli in the sequence and the last AV stimulus was not cued. Hence, the arrival of the test stimulus was unpredictable so that participants had to wait till the end of the sequence to know which stimulus was the test stimulus.

In Experiment 3 and Experiment 4, a cue consisting of a brief colour change of the fixation cross (from white to green) was presented 800 ms before the test stimulus in each sequence so that the arrival of the test stimulus was predictable.

#### 2.2 Dynamic conditions of Experiment 1 & Experiment 3

In Experiment 1 and 3, AV intervals in the adaptation sequence were either decreasing or increasing following classic hysteresis protocols using the *modified method of limits* [8]. This method was specifically designed to avoid potential decisional biases present in the traditional method of limits [27]. In particular, this method avoids the “perseveration in response bias” by randomizing ascending and descending trials as well as the “inference production from trial duration” by making all trials of the same length ([8]; see below).

**Fig 1:**
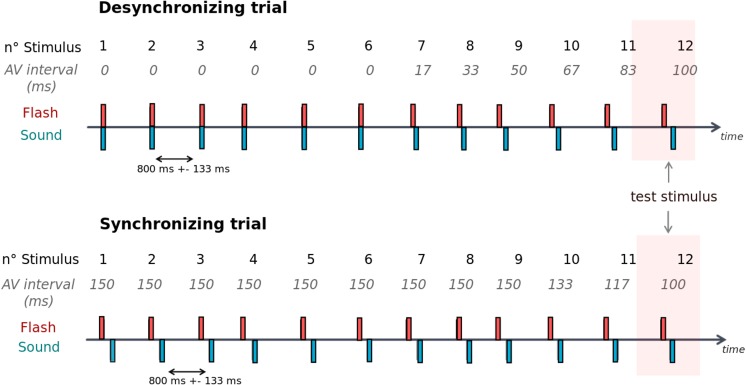
Illustration of Desynchronizing and Synchronizing trials. For illustration, the AV intervals presented here refer to flash-leads asynchronies; note that both flash-leads and sound-leads asynchronies were tested in the study. One trial was systematically composed of 12 successive AV stimuli: the first 11 stimuli consisted in the adaptation sequence and the 12th stimulus was the test stimulus. Participants were asked to report whether the test stimulus was synchronous or asynchronous. In the Desynchronizing trials (upper panel), the first AV stimulus was always synchronous and progressively desynchronized in steps of 16.7 ms until a specified AV interval at the 12^th^ position (e.g. 100 ms in this example). During the Synchronizing trials (lower panel), the first AV stimulus was always asynchronous (150 ms lag) and progressively synchronized in steps of 16.7 ms until a specified AV interval at the 12^th^ position (here, 100 ms). Crucially, the AV interval of the test stimulus (12^th^ position) was identical in Desynchronizing and Synchronizing trials although the initial interval was either 0 or 150 ms, respectively.

Three main conditions were tested. In the Desynchronizing condition, AV intervals increased in steps of 16.7 ms from synchronous to asynchronous; in the Synchronizing condition, AV stimuli were first asynchronous and AV intervals progressively decreased over time in steps of 16.7 ms (i.e. asynchrony was progressively reduced). The increment or decrement in AV intervals began at different moments across trials based on the AV interval of the 12^th^ stimulus in the sequence (see Fig. 1 and Table 1). Synchronizing and Desynchronizing sequences as well as the different test stimulus AV intervals were randomly displayed in order to exclude the response perseveration bias. In both conditions the direction of the AV interval (sound-leads or visual-leads trials) was balanced across trials.

**Table 1:** Sequences of AV intervals in Experiment 1 and Experiment 3: Synchronizing and Desynchronizing conditions.

Step	1	2	3	4	5	6	7	8	9	10	11	**12**
**Desynchronizing sequences**	0	0	0	0	0	0	0	0	0	0	0	**0**
	0	0	0	0	0	0	0	0	0	17	33	**50**
	0	0	0	0	0	0	17	33	50	67	83	**100**
	0	0	0	17	33	50	67	83	100	117	133	**150**
**Synchronizing sequences**	150	150	150	133	117	100	83	67	50	33	17	**0**
	150	150	150	150	150	150	133	117	100	83	67	**50**
	150	150	150	150	150	150	150	150	150	133	117	**100**
	150	150	150	150	150	150	150	150	150	150	150	**150**

The table shows the possible sequences of AV intervals (in ms) from the 1^st^ AV stimulus to the 12^th^ AV stimulus (“test” stimulus). All trials in Desynchronizing started with an AV interval of 0 ms; all trials in Synchronizing started with an AV interval of 150 ms. There were 12 repetitions for each test stimulus AV interval in both Desynchronizing and Synchronizing sequences; in half of the trials the sound was preceding the flash (sound-leads trials), in the other half it was the flash that preceded the sound (flash-leads trials) for a total of 12*4*2*2 or 192 trials.

#### 2.3. Constant conditions of Experiment 2 & Experiment 4

In experiment 2 and 4, a typical lag adaptation paradigm was used, namely: the Synchronizing and Desynchronizing sequences were replaced with sequences of 11 AV stimuli with a fixed time lag between the sound and the flash followed by the test stimulus (Table 2). In the 0 ms AV interval adaptation (Synchronous condition), the tone and the flash preceding the AV test stimulus were presented synchronously; in the 150 ms AV interval adaptation (Asynchronous condition), the time lag was fixed at 150 ms. As in Synchronizing and Desynchronizing sequences the direction of the AV interval (sound-leads or visual-leads trials) was balanced across trials in both conditions.

**Table 2:** Sequences of AV intervalsin Experiment 2 and 4: 0 and 150 ms constant interval conditions.

Step	1	2	3	4	5	6	7	8	9	10	11	**12**
**Synchronous sequences**	0	0	0	0	0	0	0	0	0	0	0	**0**
	0	0	0	0	0	0	0	0	0	0	0	**50**
	0	0	0	0	0	0	0	0	0	0	0	**100**
	0	0	0	0	0	0	0	0	0	0	0	**150**
**Asynchronous sequences**	150	150	150	150	150	150	150	150	150	150	150	**0**
	150	150	150	150	150	150	150	150	150	150	150	**50**
	150	150	150	150	150	150	150	150	150	150	150	**100**
	150	150	150	150	150	150	150	150	150	150	150	**150**

The table shows the possible sequences of AV intervals from stimulus 1 to 12. There were 12 repetitions of each test stimulus AV interval in both types of sequence; in half of the trials the sound was preceding the flash (sound-leads trials), in the other half it was the flash that preceded the sound (flash-leads trials) for a total of 12*4*2*2 or 192 trials.

#### 2.4. Control condition for all four experiments

In each experiment, a Control condition was run at the end of the main task. In this condition, no adaptation sequence was presented and participants performed a typical SJ task on isolated AV stimuli. The AV intervals were the same as the test stimulus AV intervals presented in Synchronizing and Desynchronizing sequences (i.e. 0 ms (synchronous); 50, 100 and 150 ms sound-leads; 50, 100 and 150 ms flash-leads). Each AV interval was presented 12 times for a total of 12*7 or 84 trials. In this condition the direction of the AV interval (sound-leads or visual-leads trials) was also balanced across trials.

### 3. Statistical analysis

Data analysis was performed using three-ways repeated-measures ANOVA using the percentage of perceived synchrony as the dependent variable and factors of Context (3 levels: Synchronizing, Desynchronizing, and Control conditions in Experiments 1 and 3; Asynchronous, Synchronous, and Control conditions in Experiments 2 and 4), test stimulus AV interval (4: 0, 50, 100 and 150 ms) and AV order (2: sound leading, flash leading). Following significant main effects, a Tukey-Kramer multiple comparisons procedure (alpha = 0.05) was performed to assess significant differences between the different levels of the factors. When the differences between conditions were not significant, we performed Bayesian statistics for null hypothesis significance testing [28,29]. The Bayes factor indicates evidence for the null hypothesis if sufficiently low (around 1/3 or lower) [30,31].

## Results

### 1. Retrospective judgments induce hysteretic effects

#### 1.1. Experiment 1: Desynchronizing/Synchronizing sequences

**Fig 2:**
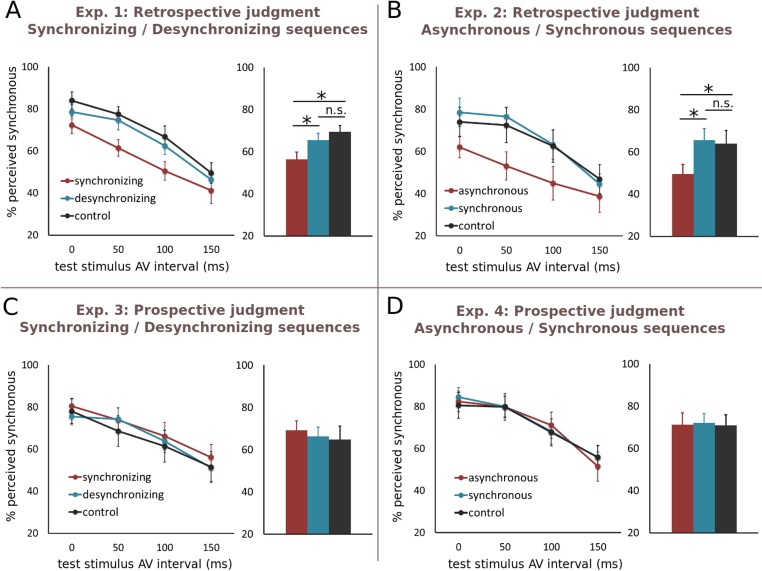
Simultaneity judgments in Retrospective and Prospective tasks. In each panel, the left graph provides the proportion of “synchronous” responses as a function of test stimulus AV interval (here, sound-leads and flash-leads trials are averaged together) and right graph plots the grand average synchronous responses across all test stimulus AV intervals for each condition. We observe hysteretic effects in the retrospective tasks (A and B). (A) Test stimuli in the Synchronizing condition (red) are perceived significantly less synchronous than stimuli in the Desynchronizing and Control condition (blue and black, respectively). No significant difference was found between the Desynchronizing and Control condition. (B) Test AV stimuli in the Asynchronous condition (red) are perceived as significantly less synchronous than stimuli in the Synchronous (blue) and Control condition (black). No significant difference was found between the Synchronous and the Control condition. In the prospective tasks (C and D), no significant effect of previous context is perceived.

Fig. 2a shows the percentage of “synchronous” responses as a function of test stimulus AV interval in the Synchronizing, Desynchronizing and Control conditions. As expected, the percentage of synchronous responses significantly decreased with increasing test stimulus AV interval (*F*_3,39_ = 28.0; *p* < 0.001; Fig. 2a). The adaptation sequence preceding the test stimulus significantly influenced Synchronous judgments (main effect of Context: *F*_2, 26_ = 10.5; *p* < 0.001). Additionally, multiple comparisons tests showed that synchrony judgments following the Synchronizing condition were significantly different from those obtained following Desynchronizing or Control conditions. More precisely, the perception of AV synchrony was overall reduced in the Synchronizing condition as compared to other conditions. These results show that AV test stimuli were perceived as being less synchronous when the initial AV stimuli in the sequence were asynchronous than when they were synchronous. This is consistent with perceptual hysteresis but inconsistent with adaptation.

However, no significant difference was observed between Desynchronizing and Control conditions: this is at odds with hysteresis predictions in which participants were predicted to report more “synchronous” responses in Desynchronizing than in Control.

The non-significant effect could reflect the absence of difference between Desynchronizing and Control conditions, but could also be due to a lack of statistical power. To differentiate between these two alternatives, we performed Bayesian statistics as classical statistics cannot disambiguate these issues [28,30,31]. We expected symmetry in hysteresis judgments, i.e. that the mean difference between Desynchronizing and Control should be of the same magnitude as the mean difference between Synchronizing and Control conditions (but in opposite directions). We thus computed the Bayes factor (BF) with an expected half-normal population distribution, with standard deviation equal to the mean difference between Synchronizing and Control conditions. The obtained BF was low (0.10), which confirmed that the Synchronous judgments did not differ between Desynchronizing and Control. Therefore, perceptual hysteresis did not seem to drive simultaneity judgments when AV stimuli are initially synchronous.

Importantly, note that neither hysteresis nor lag adaptation could account for the absence of significant effects in Desynchronizing: both would actually predict an increase in perceived AV Synchronous as compared to Control. Here, although not significant, a trend was seen in which participants tended to perceive AV stimuli as being less synchronous after Desynchronizing than in Control.

Additionally, no main effect of AV order (sound-leads or visual-leads) was observed in synchrony judgments (*F*_1,13_ = 0.4; *p* = 0.5). However, a two-ways interaction was found between order and test stimulus AV interval (interaction *F*_3, 39_ = 5.0; *p* = 0.002). This suggests that AV synchrony perception decreased more rapidly as a function of test stimulus AV intervals when the sound was leading than when it was lagging the flash. Such asymmetry is consistent with previous findings showing that perceived synchrony between sound and flash is biased toward the flash-leads asynchronies [25,26,32,33].

In summary, for Experiment 1, while synchronizing adaptation sequences biased AV synchrony towards asynchrony, desynchronizing adaptation sequences did not significantly influence AV synchrony. The present finding partly contradicts the predictions of lag adaptation as past asynchrony biased AV synchrony perception toward asynchrony (Synchronizing condition). In Experiment 2, sequences of *constant* AV lags were tested to question the role of the dynamic *versus* the constant nature of AV intervals in the generation of the reported hysteretic effect.

#### 1.2. Experiment 2: Synchronous/Asynchronous sequences

In Experiment 2, Synchronizing and Desynchronizing sequences were replaced with Synchronous and Asynchronous sequences in which the AV time lag was kept constant. Based on prior findings in the literature (e.g., [3,4]), we predicted to find adaptation effects, i.e. adapting to asynchronous stimuli with a fixed lag should lead to more synchrony reports (when the test lags are in the same direction than the adapted lag). Hence, the proportion of “synchronous” responses should be higher for Asynchronous sequences than for Control and Synchronous sequences.

As in Experiment 1, results show that the perception of synchrony significantly decreases as the temporal interval between sound and flash at the test stimulus increases (main effect of test stimulus AV interval: *F*_3,21_ = 21.4; *p* < 0.001; Fig. 2b) and that the adaptation sequence influenced synchrony perception (main effect of Context: *F*_2,14_ = 12.0; *p* < 0.001).

Surprisingly, the effect of the temporal context was very similar to the one observed in Experiment 1. Multiple comparisons test revealed that synchrony judgments following Asynchronous sequences were significantly different from those obtained after Synchronous and Control sequences: the perception of AV synchrony was overall reduced in the Asynchronous as compared to other conditions (Fig. 2b). This result is in agreement with a persistence or hysteresis effect but in disagreement with the expected adaptation after-effect [3,4] (although see [22]). In addition, Synchronous did not differ from Control (BF = 0.36) again suggesting an absence of hysteresis when the initial stimulus in the sequence is synchronous. Finally, no main effect of temporal order (sound-leads or flash-leads trials) was observed in synchrony judgments (*F*_1,7_ < 1), and no interaction between test stimulus AV interval and lag direction was observed (*F*_3,21_ < 1). Yet, the effect of context seemed to affect differently synchrony judgments depending on the test stimulus AV interval (two-ways interaction between test stimulus AV interval and context *F*_3,21_ = 6.4, p = 0.004). Specifically, temporal context impacted more synchrony judgments when the test AV intervals were ambiguous that is, close to perceptual synchrony thresholds (50 and 100 ms lags, significant multiple comparisons test).

In Experiment 2, we unexpectedly replicated a strong hysteresis effect after adaptation to steady Asynchronous sequences. This finding indicates that the persistence of asynchrony may not depend on the dynamic changes AV synchrony presentation: that is to say, the progressive synchronization of AV intervals in Experiment 1 may not be a crucial factor for perceptual hysteresis.

Hence, we hypothesized that rather than the constant *versus* dynamic nature of AV lags in the sequence, it was the retrospective task requirements in Experiment 1 and 2 that may account for perceptual hysteresis. The fact that participants were not cued for the test stimulus could have led them to evaluate individually the synchrony of each AV stimulus, although they were explicitly asked to judge the synchrony of the last stimulus in the sequence. As a consequence, participants’ synchrony judgments of the last stimulus might have been biased by their judgments on the previous AV stimuli. As will be discussed later, this could be interpreted as an updating of AV delays’ prior distribution in a Bayesian framework [13,14,34,35]. We thus designed two additional experiments using the same trials but in which a visual cue was added prior to the arrival of the AV test so that participants were aware of *when* and on which AV stimulus, they should make the simultaneity judgment.

### 2. Hysteretic effects vanished in prospective judgments tasks

#### 2.1. Experiment 3: Desynchronizing/Synchronizing sequences

Similar to the Experiment 1 and 2, a main effect of AV synchrony was found so that the perception of synchrony significantly decreased as the lag between sound and flash increased (main effect of AV lag: *F*_3,33_ = 13.5; *p* < 0.001) (Fig. 2c). Contrary to the retrospective judgment experiments, prior stimulation did not influence synchrony judgments (no main effect of Context: *F*_2,22_ < 1) (Fig. 2c).

#### 2.2. Experiment 4: Synchronous/Asynchronous sequences

Similar to Experiment 3 (Experiment 4, Fig. 2d), a significant effect of test stimulus AV interval was found (*F*_3,36_ = 32.3; *p* < 0.001). However, synchrony judgments did not differ across the different adaptation conditions (*F*_2,22_ < 1). We observed a main effect of order (*F*_1,12_ = 7.7, *p* = 0.02), suggesting that participants perceived more synchrony for the flash-leads asynchronies. In addition, a significant two-ways interaction between test stimulus AV interval and order (*F*_3,36_ = 12.2, p<0.001) suggests that large visual-leads delays were seen more synchronous than large sound-leads delays.

### 3. Retrospective vs. prospective simultaneity judgments

In order to compare the effects of the task demands (retrospective vs. prospective) and the constant *versus* dynamic nature of AV intervals in the adapting sequence on synchrony judgments, we performed an additional four-ways repeated-measures ANOVA on the data drawn from all 4 experiments with synchrony judgment as dependent variable, Participants as random factor, Context (3 levels: Initially Asynchronous (conflating Synchronizing and Asynchronous), Initially Synchronous (conflating Desynchronizing and Synchronous) and Control), test stimulus AV interval (4 levels: 0, 50, 100, 150 ms) and order (2 levels: sound-leads, flash-leads)) as fixed factors, and the between-groups factors Task (2 levels: retrospective and prospective) and sequence type (2 levels: dynamic or constant).

As expected, the analysis showed a main significant influence of test stimulus AV interval in simultaneity reports (*F*_3,123_ = 88.9; *p* < 0.001). While the proportion of “synchronous” responses was not significantly influenced by the sequence type of stimulus (*F*_3,123_ < 1) or by the task (*F*_1,41_ = 3.2; *p* = 0.08), we observed a two-ways interaction of task and context (*F*_2,82_ = 10.1; *p* < 0.001). This significant interaction suggests that the task has a crucial impact on synchrony reports. The task specifically influenced the perceived synchrony following Initially Asynchronous sequences, namely: hysteretic biases were found when participants were asked to judge AV synchrony retrospectively after the presentation of the test stimulus, but these biases were not observed in prospective judgment tasks.

## Discussion

This series of experiments provides two novel findings in the perception of AV simultaneity: first, the presence of hysteretic effects and second, the impact of retrospective *vs*. prospective decisions. Experiment 1 and 2 showed that persistence or hysteresis effects only occurred when the adaptation sequence was initially asynchronous (Synchronizing in Experiment 1 and Asynchronous in Experiment 2): while participants showed a strong tendency to persist in their perception of AV asynchrony, they did not persist in their perception of AV synchrony. In addition, we showed that the presence of persistence effects may not depend on the dynamic nature of the adaptation period, but rather on the nature of task demands: perceptual hysteresis occurred for retrospective tasks (Experiment 1 and 2), and was not seen for prospective tasks (Experiment 3 and 4).

While hysteretic effects have been reported in tactile [18] and audiovisual [15] TOJ tasks, our results provide the first evidence that SJs can also exhibit hysteresis. However, TOJs and SJs entail distinct psychological processes [36–39] and more specifically distinct decisional biases [23,37]. Our results and previous reports [3,4,15] thus show that TOJs and SJs share at least the propensity to be biased by past context, either towards lag adaptation or perceptual hysteresis.

The presence of hysteretic and adaptation biases in AV TOJs and SJs could be due to a change in the encoding of perceptual timing, or to a change of decisional criteria induced by task experimental manipulation (i.e., retrospective vs. prospective judgments). The perceptual or decisional origin of lag adaptation effects is still debated [22,40–43]. The present findings, for their part, support a decisional origin of AV timing hysteresis. The disappearance of hysteretic effects observed in experiments 3 and 4 are consistent with the proposal that task manipulation entails judgmental comparisons [44]. In other words, ambiguity in synchrony judgments is likely not generated by the competition between clear bistable percepts, but is rather caused by uncertain decisions. This suggests that simultaneity and successiveness may not be perceptually categorical but only distinguishable at a later decisional level, at least for short lag durations.

Additionally, in recent studies, both adaptation (aftereffects) and hysteresis have been interpreted within a Bayesian framework [13,34,35]. In a Bayesian account of perceptual hysteresis, the priming of one of the two possible perceptual outcomes or alternative states (here, asynchronous vs. synchronous) adjusts the previous knowledge (i.e. the prior) towards that perceptual state [13,14]. In contrast, adaptation causes a reduction of the sensory evidence for the adapted stimulus (i.e., the probability density of the likelihood function is reduced) [13,14,34,35]. Similarly, our results could be interpreted within the Bayesian framework as a drift over time of the prior bias in synchrony judgments [14].

First, the retrospective *vs*. prospective nature of the task may be crucial in privileging the adjustment of the prior (or hysteresis) over the adjustment of sensory evidence (or lag adaptation). In retrospective tasks (Experiment 1 and 2), participants were explicitly asked to only judge the synchrony of the last stimulus. Although they were told that the timing of past stimuli was irrelevant to perform the task, participants may have individually evaluated or monitored every AV stimulus in the adaptation sequence. This could have led to the updating of the internal prior at each stimulus presentation. The Bayesian framework could thus account for these results if we assume that the default prior for AV synchrony has been set to ‘synchronous’: the adjustment of the prior from ‘synchronous’ to ‘asynchronous’ after each stimulus predicts the decrease of synchrony perception observed in Synchronizing (Experiment 1) and Asynchronous (Experiment 2) conditions; and the default “AV synchrony” prior predicts also the absence of contextual effect after Desynchronizing (Experiment 1) and Synchronous (Experiment 2) sequences. Second, in Experiment 3 and 4, participants may wait for the cue before emitting AV timing judgments, and as such may not modify their prior knowledge during the presentation of the sequence. In these conditions, the contextual effects should only operate with the updating of sensory evidence. Past studies having shown these effects used long periods of adaptation (several minutes) [3,4]. Here, the adaptation phase might have been too short to entail lag adaptation (even though recent reports have suggested that lag adaptation occurs rapidly [45]). We thus propose that the presence of hysteresis *vs*. adaptation effects in our experiment originates from the retrospective *vs*. prospective task.

An alternative attentional account could be argued, namely: although participants were instructed to focus their attention over the entire adaptation sequence, participants paid overall more attention to AV timing in retrospective experiments due to uncertainty in temporal expectation. However, prior studies have shown that attention to AV timing tends to increase lag adaptation effects compared to passive viewing [17]. Here, no significant lag adaptation effects were observed.

Furthermore, an attentional interpretation cannot account for the difference in simultaneity reports between Synchronizing and Desynchronizing sequences (Experiment 1) or between Asynchronous and Synchronous sequences (Experiment 2). If participants paid more attention to the stimuli they would be overall more “focused” on the task and be more precise in their judgments [46]. Along this line of argument, the drop in synchrony reports in the retrospective judgments could be due to a better detection of small AV asynchrony lags. Accordingly, the global attentional effect should not be specific to the type of sequence presented to the participants and similar synchrony judgments should be observed in both Synchronizing and Desynchronizing conditions, and in both Asynchronous and Synchronous conditions. However, this is not what we found.

Finally, one could also argue that the predictive argument fails to the extent that the test stimulus was always at the 12^th^ position in the sequence. Although participants were not explicitly told about this regularity, some participants may have noticed it and consciously counted the number of items in the sequence to predict the arrival of the test stimulus. However, this cannot account for the findings either: if counting had a global effect in distracting or helping the participant in the task, the same resulting bias should be observed for all sequence conditions. Again, the difference in simultaneity reports between Synchronizing/Desynchronizing and Asynchronous/ Synchronous conditions rules out this hypothesis.

It has been argued [9,13,47] that the dual effect of hysteresis and adaptation is necessary for exploiting past sensory information in an optimal fashion: while hysteresis stabilizes perceptual states against continuously changing low-level sensory data, adaptation biases towards the analysis of new information. Thus, the interplay between hysteresis and adaptation in AV perception can open new research venues. For instance, in AV timing, adaptation is typically associated with the optimization of AV integration which can compensate for slight natural AV asynchronies and improve the binding of auditory and visual information [3,4,48]. However, ecological AV timing is sometimes uncorrelated, for instance in an environment with multiple speakers: as illustrated in the cocktail party phenomenon, an individual may hear one speaker while observing another speaker talking and require attentional shift to realign congruent AV inputs. In such ecological situation, binding asynchronous stimuli would have detrimental consequences on perception, e.g. not understanding one speaker’s utterance [49,50]. In natural situations such as in AV speech, hysteresis could help maintain apart the processing of irrelevant AV information despite the transient sensory evidence of AV synchrony—in other words, hysteresis would help solving the AV cocktail party effect. Hence, hysteretic effects highlight the impact of possible top-down and predictive coding in AV integration: bottom-up (a)synchrony may not be as crucial as the second order temporal statistics shared by high level representations, and which have been shown to drive supramodal processing [51–53].

It is also noteworthy that while the present study has focused on the effect of past context on AV temporal judgment tasks, we speculate that these effects could apply to a broader range of perceptual decisions. In particular, hysteretic effects in vision and in audition were usually reported when participants did not know when to estimate the stimulus of interest [7–12], while in most visual/auditory adaptation studies participants were cued to the arrival of the test stimulus [3,4,16–19,54]. We contend that prospective and retrospective tasks may be an important differing factor in adaptation and hysteresis paradigms.
